# *Giardia lamblia-*infected preschoolers present growth delays independent of the assemblage A, B or E

**DOI:** 10.1590/0074-02760230043

**Published:** 2023-08-18

**Authors:** Maria Fantinatti, Tiara Cascais-Figueredo, Phelipe Austriaco-Teixeira, Filipe Anibal Carvalho-Costa, Alda Maria Da-Cruz

**Affiliations:** 1 Universidade Federal de Roraima, Curso de Medicina, Boa Vista, RR, Brasil; 2 Fundação Oswaldo Cruz-Fiocruz, Instituto Oswaldo Cruz, Laboratório Interdisciplinar de Pesquisas Médicas, Rio de Janeiro, RJ, Brasil; 3 Fundação Oswaldo Cruz-Fiocruz, Instituto Oswaldo Cruz, Laboratório de Epidemiologia e Sistemática Molecular, Rio de Janeiro, RJ, Brasil; 4 Universidade do Estado do Rio de Janeiro, Faculdade de Ciências Médicas, Disciplina de Parasitologia, Rio de Janeiro, RJ, Brasil

**Keywords:** Giardia lamblia, height for age, weight for age, pre-schoolers, genotyping

## Abstract

**BACKGROUND:**

Intestinal parasite *Giardia* can affect children’s physical development mainly stunting even in asymptomatic cases. The protozoa *G. lamblia* is divided into assemblages A-H. However, it is still unclear whether clinical manifestations and pathogenesis may vary according to the infecting assemblage.

**OBJECTIVES:**

To investigate whether *G. lamblia* assemblages influence differently the physical development of preschoolers from a community of Rio de Janeiro, Brazil.

**METHODS:**

Anthropometric parameters were analysed from children attending a daycare centre and stool samples were obtained for the *G. lamblia* diagnosis. *G. lamblia* isolates from positive samples were genotyped. Data were analysed in order to verify whether there is a relationship between *G. lamblia* infection and the physical development of children according to the assemblage.

**FINDINGS:**

Herein we demonstrated that although eutrophic, *G. lamblia*-infected daycare preschoolers from a low-income community presented growth delay compared to non-infected ones. This effect was observed for the three assemblages (A, B or E) found infecting humans.

**MAIN CONCLUSION:**

*G. lamblia* causes growth delays on children independent of infecting assemblage (A, B or E).

*Giardia lamblia* is the most common flagellate intestinal protozoan in human infections. The species is phylogenetically divided into assemblages from A to H.^1^ The assemblages A and B present a high zoonotic potential and are classically reported in humans.^1^ The other assemblages (C-H) are considered host-specific, however this affinity for the host is discussed because the circulation of these assemblages in atypical hosts has grown.^2^ ^,^ ^3^ The assemblage E is associated with infection in farm animals, however several reports of human infection were reported.^2^ ^,^ ^4^ ^,^ ^5^ ^)^ Although the genetic divergences of these assemblages and their affinity for groups of hosts are known, there is still a lack of knowledge if the pathogenesis, clinical manifestations or drug resistance predisposition can vary according to the infecting assemblage.

Infection by assemblages A and B can be asymptomatic or show a wide spectrum of clinical features and may vary depending on the characteristics of the population evaluated.^6^ ^-^ ^13^ The clinical presentation directly related to assemblage E in human infection has not yet been reported. There are growing evidences that giardiasis impact on children development. *G. lamblia-*infected children present growth delays and weight reduction, even without apparent clinical symptoms as diarrheal disease.^14^ ^-^ ^20^

Malnutrition is commonly observed in *G. lamblia-*infected children,^21^ who present reduced weight, serum iron and zinc levels.^17^ Malabsorption is reported in at least 50% of symptomatic giardiasis patients,^14^ ^-^ ^16^ which can cause growth delays of up to 4 months after an episode of diarrhoea.^14^ ^,^ ^15^ However, most studies looking at the physical impacts associated with *G. lamblia* infection are carried out with individuals from areas of social vulnerability where other factors such as malnutrition associated with economic conditions may represent confounding factors.^22^ ^,^ ^23^ It is unknown whether *G. lamblia* infection can also harm apparently healthy and asymptomatic children in subclinical infections and whether the impact of infection is related to the infecting assemblage. In this context, this study aims to investigate whether *G. lamblia* assemblages differently affect the physical development of asymptomatic preschoolers attended at a municipal daycare centre located in a low-income community of Rio de Janeiro, Brazil.

## MATERIALS AND METHODS

*Study design, collection of faecal samples and parasitological examinations* - A cross-sectional survey was carried out in the years 2014 and 2015 in a public municipal daycare in a low-income community of Rio de Janeiro. The daycare hosts children aged 10 months to 4 years, for a period of 9 hours (7:30 am to 16:30 pm), and provides five meals balanced by nutritionist a day. The demographic information regarding sex and age (months) were also collected.

A single stool sample was collected from 194 out 220 subjects. For the diagnosis of geohelminths and protozoa, the samples were submitted to parasitological examination by the Ritchie method. In parallel, stool aliquots were conditioned at -20^o^C until molecular diagnosis and genotyping.

All children infected with intestinal parasites were monitored by physicians from the Brazilian program Saúde da Família (PSF) at Centro de Saúde Heitor Beltrão, Secretaria Municipal de Saúde, Prefeitura da Cidade do Rio de Janeiro and were treated with medications recommended by the Brazilian Ministry of Health.

*Ethics* - The subjects were included in the research after responsible agreement and of the free and informed consent form signature, according to the ethical requirements. This study was approved by the ethics review board in human research from Instituto Oswaldo Cruz, Fundação Oswaldo Cruz-Fiocruz (CAAE 24712319.6.0000.5248).

*Molecular diagnosis and genotyping of G. lamblia* - DNA extraction from faeces was performed using the QIAamp DNA Stool Mini Kit (Qiagen GmbH, Hilden, Germany), according to the manufacturer’s instructions, with the exception of the lysis temperature, which was increased to 95°C, and the volume of AE buffer used for DNA elution, which was decreased to 100 μL. The isolated DNA was stored at -20°C until the time of use.

For the diagnosis and genotyping of *G. lamblia*, the conserved genes coding for the protein glutamate dehydrogenase (*gdh*) and beta-giardin (*βgia*) were used, as described by Fantinatti et al.^24^

*Anthropometric measurements* - Anthropometric data (weight and height) of all children included were obtained after the completion of stool sample collection and before treatment. The measurements were taken at the daycare centre with the support of a nursery team of the local municipal health department (PSF), in a single moment per year. The weight was measured in the range of 0.01 kg using a digital scale and height in the nearest 0.1 cm using with a millimetrered tape. Standard deviation scores (Z-scores) of weight-for-height (WHZ), height-for-age (HAZ) and weight-for-age (WAZ) were calculated using the NutStat Module on EpiInfo 2000 (according to Centers for Disease Control and Prevention, Atlanta, USA) and the World Health Organization’s 1978 growth chart. The Z-score corresponds to a dispersion measure with standard deviation (SD) in a reference population. Stunting, wasting and underweight were defined by -2 SD from mean HAZ, WHZ and WAZ, respectively.^25^

*Statistical analyses* - The statistical analyses were performed using GraphPad Prism software (version 6.0, San Diego, USA). The Z-scores of positive-infected individuals with distinct *G. lamblia* assemblages and negative-infected individuals were compared using the Mann-Whitney *U* non-parametric test. Statistical significance was established at p < 0.05.

## RESULTS

*Frequency of G. lamblia infection and their assemblages among pre-schoolers* - A total of 194 faecal samples were collected from children, of which 86 samples (44.3%) were positive for *G. lamblia*. Regarding gender, there was no statistical difference among *Giardia* infected preschoolers, infection rate reaching 51/109 (46.8%) in females and 35/85 (41.2%) in males (p = 0.7).

In addition to *G. lamblia*, the stool examination revealed the following intestinal parasites and their respective frequencies: *Entamoeba histolytica*/*dispar*/*moshkovskii*/*bangladeshi* (4/194 - 2.06%), *Entamoeba coli* (5/194 - 2.58%), *Endolimax nana* (13/194 - 6.70%) and *Ascaris lumbricoides* (15/194 - 7.73%). 

*G. lamblia*-positive stool DNA samples were extracted and the parasite was genotyped using *gdh* and *βgia* markers with the following distribution: 42 (48.8%) assemblage A, 21 (24.4%) assemblage B, 19 (22.1%) assemblage E and four (4.7%) assemblage A/E (Supplementary Table).^24^ There were no differences in assemblages frequencies through genotyping with distinct markers.

*G. lamblia infection and children’s nutritional status* - The anthropometric parameters were evaluated in 167 children. The vast majority of children was classified as eutrophic for HAZ (n = 130), WAZ (n = 152) and WHZ (n = 154).

Although eutrophic for the HAZ and WAZ parameters, children infected by *G. lamblia* had significantly lower Z-score medians (interquartile range) (-1.36 [-1.79 to -0.48], n = 60 and -0.35 [-1.36 to +0.34], n = 71) (A in Figure) values than non-infected ones (-0.47 [-1.10 to -0.11], n = 81 and 0.02 [-0.58 to -0.53], n = 96), p = 0.0001 and p = 0.017, respectively (C in Figure). For the WHZ parameter, there was no difference in Z-score between groups (*Giardia*-positive: 0.47 [+0,42 to +0,93] n = 71, *Giardia*-negative: 0,55 [-0,20 to +0,94] n = 95), p = 0.65 (E in Figure) (Supplementary Table).

*G. lamblia* infected children were subdivided according to the infecting assemblage (A, B or E). Individuals infected with any of the three *G. lamblia* assemblage A, B or E showed significantly lower HAZ score values compared to *Giardia*-negative individuals (p = 0.03, p = 0.03, p = 0.003, respectively) (B in Figure). No difference was seen for HAZ score values among the subgroups of children infected by the different assemblages. In addition, although *G. lamblia* positive children did present lower weight, no significant difference was observed for WAZ among individuals infected by assemblages A, B and E compared to the group of *Giardia*-negative (D in Figure). Regarding the WHZ parameter, no differences were observed between the group *Giardia*-negative children compared with the subgroups of children infected by the different assemblages (F in Figure) (Supplementary Table).

**Figure:**
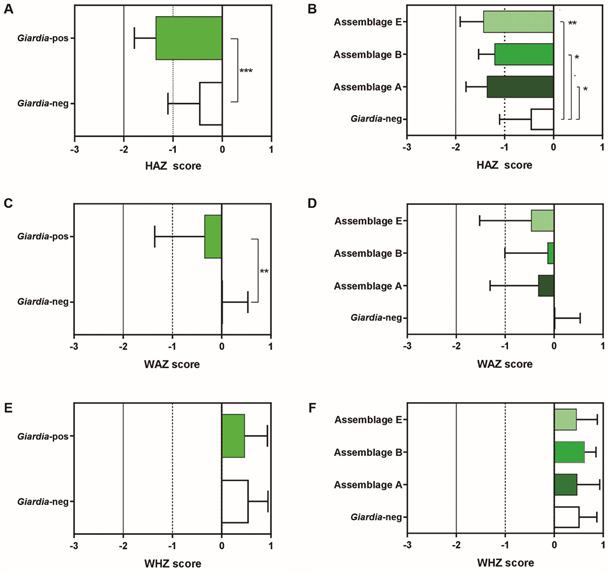
Influence of *Giardia lamblia* infection and its assemblages on anthropometric parameters measured on preschool children. A and B: height-for-age (HAZ); C and D: weight-for-age (WAZ); E and F: weight-for-height (WHZ). The insert in A, C and E is a dot plot representation of individual’s Z-score values. The column bar represents the median values with interquartile range. Asterisks denote statistically significant differences between groups compared by Mann-Whitney *U* non-parametric test. * p < 0.05; ** p < 0.005. *Giardia*-pos: *Giardia*-positive; *Giardia*-neg: *Giardia*-negative.

## DISCUSSION

In this study, a high infection rate of *G. lamblia* was demonstrated, with almost half of the children in the daycare centre evaluated as positive for this infection, as well as a high genotypic diversity of the parasite, in which A, B and E assemblages were detected. The main finding was the influence of *G. lamblia* infection on the nutritional parameters of children, specifically HAZ and WAZ. The malabsorptive syndrome induced by *G. lamblia* can cause nutritional deficits and growth faltering in childhood as a result of chronic damage, even in asymptomatic individuals.^18^ ^-^ ^20^ ^,^ ^26^ ^,^ ^27^

The biological properties, besides distinct antigenic composition of different *G. lamblia* assemblages, can affect the parasite-host relationship, possibly accounting to the clinical diversity of the infection.^3^ In the present study, we evaluated putative differences in *G. lamblia* assemblage’s pathogenicity showing that, despite eutrophic, children infected by *G. lamblia* presented growth retardation regardless the assemblage (A, B or E) they were infected.

In Brazil, a significant reduction in malnutrition besides no increase in overweight cases have been observed in children under 5 years of age.^28^ The preschoolers studied herein stay in the daycare 9-hours by day, where they have a balanced nutritional support (five meals). Therefore, as expected, most children were eutrophic. It points that the physical impairment observed herein is probably a direct consequence of the parasite infection, as already proven in a murine experimental model.^29^ In this study, the nutritional indicators influenced by *G. lamblia* infection were HAZ and WAZ, confirming previous results evidencing an insidious effect of subclinical infections, not associated with acute or persistent diarrheal disease.^20^

Experimental model studies have shown that *Giardia* infection can lead to changes in small intestine physiology, causing disruption of tight junctions, enterocyte apoptosis, microvilli shortening, altered trypsin activity of enterocyte and interfering with Na/Cl and in the metabolism of glucose and minerals.^30^ Interference in biliary activity impairs lipid metabolism leading, consequently, to the reduction of fat-soluble vitamins (A, D, E and K). These changes may be associated with deficits in deficits in children’s development, especially with the reduction in HAZ indices.

*G. lamblia*-associated growth delays have also been observed in others studies.^19^ ^,^ ^31^ ^-^ ^35^ This is in line with the hypothesis that *G. lamblia* is one of the major contributors to decrement on length in young children.^23^ ^,^ ^32^ Whether any assemblage of *G. lamblia* is faster to induce intestinal damage, impair nutritional intake and consequently physical development is unknown.

All the three *G. lamblia* assemblages found infecting humans (A, B and E)^2^ ^,^ ^4^ were detected in our previous study area.^24^ ^)^ Importantly, there was no specific assemblage associated with decrease in length.

Many studies have been carried out in an attempt to associate assemblages A and B with a symptom or a set of symptoms. However, the studies show divergence between the findings, suggesting that this association is multifactorial, and should be considered characteristics of the parasite, host and environment (endemic strains and newly introduced strains).^3^ There are reports in the literature of the assemblage E infecting humans but, as these findings are recent, the association of this genotype with the pathogenesis of human giardiasis is still unknown.^4^ ^,^ ^5^ ^,^ ^24^ ^,^ ^36^ ^-^ ^40^

We also identified the presence of other pathogenic intestinal parasites (*A. lumbricoides* and *E. histolytica*) that could also impair the physical development of these children.^41^ ^-^ ^45^ As previous results have already shown an association between *Giardia* infection and growth impair,^23^ besides the high frequency of this parasite in our casuistic, we decided to investigate it.

In a limiting way, this was a cross-sectional study and, therefore, it is not possible to exclude the possibility of reverse causality. In other words, the low incoming socioeconomic conditions observed in rural areas and slums could favour inadequate nutrition for these children, which could increase the risk of infection. The preschoolers studied herein present a high frequency (44.3%) of *G. lamblia* infection and are exposed to frequent reinfections.^24^ Chronic parasitism leading to inflammatory disorders of the proximal small intestine, condition known as environmental enteric dysfunction,^46^ can be related to *G. lamblia* infection and underlies growth faltering in our study population.

In conclusion, *G. lamblia* is associated with deficits in physical development in pre-school children living the study area, independently of the infecting assemblage. Giardiasis should be targeted by specific control measures, with improved diagnosis at the community level and access to treatment.
