# Supplementary material for: Giardia lamblia-infected preschoolers present growth delays independent of the assemblage A, B or E
Source: Mem Inst Oswaldo Cruz. 2023 Aug 18;118:e230043. doi: 10.1590/0074-02760230043 (PMC10443202; doi:10.1590/0074-02760230043)
Supplement: Supplementary file 1 [file 1678-8060-mioc-118-e230043-s.pdf]

TABLE

Demographic and parasitological data of the children followed up in the study

Sample identification	Gender	Age in months	Diagnosis	Assemblage	HAZ	WAZ	WHZ
1	M	36.9	*Ascaris lumbricoides*		-0.66	0.29	0.89
2	F	22.1	Negative		No	-0.78	-1.46
3	F	32.0	Negative		-0.62	-0.07	0.38
4	F	20.7	Negative		No	-1.18	-1.37
5	F	42.2	*Giardia lamblia*	A	-1.84	-2.62	-2.07
6	F	12.7	Negative		No	-1.13	No
7	M	22.4	Negative		No	1.21	0.47
8	F	51.3	Negative		0.41	0.32	0.24
9	M	37.4	*Giardia lamblia*	A	-1.57	0.19	1.45
10	M	33.9	Negative		0.10	-0.10	-0.28
11	M	47.4	*Entamoeba histolytica/díspar/moshkovskii/bangladeshi*		0.03	0.14	0.12
12	M	40.9	*Giardia lamblia*	A	-0.26	0.52	0.87
13	M	29.3	*Ascaris lumbricoides*		0.94	-0.13	-0.97
14	M	38.0	Negative		-0.58	0.19	0.68
15	M	40.5	Negative		-0.46	0.21	0.60
16	M	37.4	Negative		-0.75	0.23	0.87
17	M	51.9	Negative		-0.10	0.54	0.86
18	M	44.3	*Giardia lamblia*	E	-0.76	0.07	0.68
19	M	18.2	*Giardia lamblia*	A	No	-0.27	0.01
20	F	35.6	Negative		-1.47	-0.39	0.65
21	M	30.4	Negative		-0.62	-0.51	-0.18
22	F	51.8	Negative		0.68	-0.16	-0.79
23	F	41.1	Negative		-0.20	0.22	0.52
24	F	54.1	Negative		-0.49	-0.12	0.40
25	F	28.0	*Giardia lamblia*	E	-1.20	-0.13	0.88
26	M	45.2	Negative		-1.13	0.66	0.66
27	M	22.0	*Giardia lamblia*	A	No	-2.20	-0.63
28	F	37.3	Negative		-0.17	0.91	1.41
29	F	38.6	Negative		-0.10	0.37	0.63
30	M	45.7	*Endolimax nana*		0.27	0.29	0.12
31	M	30.9	*Giardia lamblia*	A	-1.83	2.88	5.04
32	F	30.9	*Giardia lamblia*	E	-1.76	-1.36	-0.34
33	F	25.3	*Giardia lamblia*	A	0.26	0.22	0.25
34	M	34.5	*Giardia lamblia*	A	-0.81	0.39	1.16
35	M	26.4	*Giardia lamblia*	A	-1.79	-2.32	-1.54
36	F	50.8	*Endolimax nana*		0.47	0.31	0.18
37	F	22.6	*Giardia lamblia*	E	No	-0.11	0.31
38	F	43.2	*Giardia lamblia*	E	-1.97	-2.72	-2.07
39	F	31.7	*Giardia lamblia*	E	-1.90	-0.50	0.88
40	F	23.9	*Giardia lamblia*	A	No	0.69	0.68
41	F	41.7	*Giardia lamblia*	A	-1.79	-1.90	-1.11
42	M	40.2	*Giardia lamblia*	E	-0.93	0.25	1.04
43	F	36.1	*Giardia lamblia*	A	-1.02	-1.38	-1.15
44	F	45.4	*Giardia lamblia*	A	1.09	1.20	0.93

Sample identification	Gender	Age in months	Diagnosis	Assemblage	HAZ	WAZ	WHZ
45	M	54.3	*Ascaris lumbricoides*<br>*Entamoeba histolytica/díspar/moshkovskii/bangladeshi*		-0.62	0.03	0.62
46	F	30.1	*Giardia lamblia*	A	-1.08	-0.35	0.43
47	F	63.7	*Endolimax nana*		-1.45	-0.83	0.27
48	F	33.6	*Ascaris lumbricoides*		-0.12	0.81	1.20
49	F	36.8	*Ascaris lumbricoides*<br>*Endolimax nana*		-0.10	0.56	0.87
50	M	22.5	Negative		1.67	2.15	1.57
51	M	49.7	*Giardia lamblia*	A	-2.16	-0.60	1.04
52	F	33.6	*Giardia lamblia*	E	-1.43	-1.77	-1.27
53	M	38.5	*Giardia lamblia*	A	0.12	0.73	0.87
54	M	31.0	*Giardia lamblia*	A	-1.85	2.28	4.35
55	F	51.7	*Giardia lamblia*	A	0.26	0.64	0.83
56	F	21.3	*Giardia lamblia*	A/E	No	1.13	0.82
57	F	58.9	*Giardia lamblia*	A	-1.54	-1.67	-0.85
58	F	25.9	Negative		-0.18	-1.17	-1.33
59	F	44.7	Negative		-0.29	0.19	0.55
60	M	64.5	*Giardia lamblia*<br>*Ascaris lumbricoides*	A	-1.31	-0.75	0.29
61	M	29.2	*Giardia lamblia*	A	-3.82	-3.07	-0.70
62	M	51.5	*Giardia lamblia*	A	-1.89	-0.35	1.18
63	M	50.5	Negative		-0.15	0.42	0.72
64	F	25.4	Negative		-0.21	-1.12	-1.20
65	F	38.0	*Ascaris lumbricoides*<br>*Entamoeba histolytica/díspar/moshkovskii/bangladeshi*				
66	F	21.1	Negative		No	-2.27	-1.41
67	F	47.5	Negative		-0.81	-0.73	-0.27
68	F	25.3	Negative		0.44	1.82	2.29
69	M	30.1	*Giardia lamblia*	E	-1.94	-1.51	-0.41
70	M	50.6	Negative		-0.16	0.61	1.00
71	M	22.7	Negative		No	0.55	0.93
72	M	33.2	*Ascaris lumbricoides*<br>*Giardia lamblia*	A	-1.69	-0.64	0.47
73	F	40.4	*Giardia lamblia*	E	-2.38	-1.80	-0.42
74	F	28.8	*Giardia lamblia*	A	0.26	-1.27	-2.07
75	F	21.9	Negative		No	1.03	1.45
76	F	29.5	*Giardia lamblia*	E	-0.97	0.76	1.84
77	F	40.1	*Giardia lamblia*	A	-0.31	-0.83	-0.98
78	F	18.7	Negative		No	-0.94	-1.36
79	F	45.9	*Giardia lamblia*	E	-0.60	-0.47	-0.09
80	M	25.8	*Giardia lamblia*	A	-1.08	-0.30	0.68
81	F	47.4	Negative		1.26	0.24	-0.70
82	F	21.3	*Giardia lamblia*	A	No	0.08	-2.54
83	M	35.5	Negative		0.35	1.13	1.25
84	M	39.5	Negative		0.70	1.63	1.64
85	F	31.0	*Giardia lamblia*	A/E	-1.78	-0.44	0.88
86	F	40.8	*Giardia lamblia*	A	-1.41	-1.76	-1.31
87	F	42.1	*Giardia lamblia*	E	-2.35	-2.14	-0.89

Sample identification	Gender	Age in months	Diagnosis	Assemblage	HAZ	WAZ	WHZ
88	F	42.8	*Ascaris lumbricoides*		-2.69	-0.57	1.48
89	M	26.9	*Giardia lamblia*	E	-1.88	-1.52	-0.34
90	M	23.9	*Endolimax nana*		No	-0.71	-0.89
92	M	48.9	Negative		-0.17	0.12	0.12
93	F	34.1	*Giardia lamblia*	B	-0.98	-0.04	0.72
94	F	28.1	*Giardia lamblia*<br>*Ascaris lumbricoides*	A			
96	F	23.7	Negative		No	2.02	2.29
98	F	34.9	Negative		-1.10	-0.09	0.75
99	F	25.0	*Giardia lamblia*	E			
100	F	29.6	*Giardia lamblia*	B			
101	F	32.7	*Giardia lamblia*	B			
102	F	54.2	Negative		-0.50	-0.13	0.40
103	F	36.7	*Giardia lamblia*	E	-0.34	1.64	2.49
104	F	34.0	*Giardia lamblia*	B	-1.22	No	No
105	F	23.0	Negative		No	0.89	1.88
106	F	32.3	Negative				
107	M	34.4	*Giardia lamblia*	B	-1.62	-0.47	0.63
108	M	38.4	Negative		0.88	1.68	1.57
109	F	30.7	Negative		-1.45	-0.19	0.94
110	F	52.8	*Entamoeba coli*		-1.24	0.55	1.83
111	M	46.1	*Giardia lamblia*	B	-1.48	-0.57	0.42
112	F	27.3	*Giardia lamblia*<br>*Entamoeba coli*<br>*Ascaris lumbricoides*	A	-0.22	0.31	0.68
114	M	27.3	Negative				
116	M	52.9	Negative		-0.22	0.89	1.45
117	M	30.8	Negative		0.11	0.21	0.21
118	M	20.2	*Giardia lamblia*	A	No	-0.53	0.01
119	M	34.3	*Entamoeba coli*<br>*Ascaris lumbricoides*		-2.17	-2.21	-1.30
120	M	39.7	Negative		-1.11	0.02	0.87
121	M	41.3	Negative				
122	M	52.5	*Giardia lamblia*	B	-1.55	-0.12	1.21
124	F	38.1	Negative		-0.29	0.84	1.41
125	F	29.0	*Giardia lamblia*	E	-1.40	-0.23	0.88
126	M	21.1	Negative				
127	F	20.2	*Giardia lamblia*	A	No	-2.62	-1.72
128	M	30.2	*Ascaris lumbricoides*		-1.97	0.32	2.04
129	F	47.6	*Giardia lamblia*	B			
130	F	45.7	Negative				
131	M	42.4	*Giardia lamblia*	B			
133	F	35.4	Negative		-2.76	-1.25	0.62
134	F	32.7	Negative		-1.81	-0.37	0.96
135	M	35.9	Negative		-2.17	-1.39	-0.21
136	F	28.4	*Giardia lamblia*<br>*Entamoeba coli*	A			

Sample identification	Gender	Age in months	Diagnosis	Assemblage	HAZ	WAZ	WHZ
137	M	30.5	Negative		-0.36	0.51	1.04
138	M	34.0	*Giardia lamblia*	A	1.35	1.74	1.29
139	F	44.0	Negative		-0.12	0.17	0.42
141	F	50.6	Negative				
143	M	46.7	Negative		-0.60	0.65	1.37
144	M	27.1	Negative		1.93	0.73	-0.43
145	F	42.9	Negative		-0.69	-0.63	-0.29
146	M	43.7	*Endolimax nana*<br>Ascaris lumbricoides<br>Entamoeba histolytica/díspar/moshkovskii/bangladeshi		-1.93	-0.35	1.07
147	M	38.4	*Giardia lamblia*	A	-1.45	0.05	1.18
148	M	27.9	Negative				
149	F	32.3	*Giardia lamblia*	A	-1.47	0.49	1.84
151	F	39.3	Negative		-0.20	0.31	0.63
152	F	35.9	*Giardia lamblia*	B	-0.23	-0.13	-0.04
153	F	53.8	*Giardia lamblia*<br>*Endolimax nana*	A	-0.01	0.56	0.93
154	F	34.6	*Giardia lamblia*<br>*Ascaris lumbricoides*	A	-1.58	-1.24	-0.42
155	M	52.2	Negative		-0.14	0.51	0.86
156	M	21.0	Negative				
157	F	48.1	Negative		-0.89	-0.79	-0.27
159	M	20.8	*Giardia lamblia*	E			
160	M	38.3	Negative		-0.62	0.17	0.68
161	M	31.7	Negative		-1.97	-1.02	0.23
162	F	20.7	Negative		No	-1.18	-1.37
163	F	36.9	Negative		-1.65	-0.58	0.56
164	F	37.2	*Giardia lamblia*	E	0.10	1.55	2.03
166	F	21.9	*Giardia lamblia*<br>*Endolimax nana*	A	No	-1.32	-0.36
167	F	48.8	Negative				
169	M	21.8	*Giardia lamblia*	B	No	-2.18	-0.63
170	F	45.6	Negative				
171	F	27.2	Negative		-0.47	-1.41	-1.46
173	M	21.6	Negative		No	-0.01	0.72
174	M	28.2	*Giardia lamblia*	A/E			
175	F	33.3	*Giardia lamblia*	B			
176	F	27.7	Negative		-0.04	-0.08	-0.01
177	M	22.4	*Endolimax nana*		No	-0.01	0.12
178	F	37.9	*Giardia lamblia*	B			
180	F	20.2	*Giardia lamblia*	A	No	-0.77	0.01
181	M	41.8	Negative		-0.64	0.10	0.60
182	M	41.2	*Endolimax nana*		-1.07	0.79	1.90
183	F	37.4	*Endolimax nana*		-0.19	-0.12	-0.03
185	F	50.0	*Giardia lamblia*	B	-0.37	0.34	0.80
186	F	37.3	Negative		-2.50	-2.42	-1.12
187	M	27.9	*Giardia lamblia*<br>*Endolimax nana*	B	-1.24	-1.44	-0.86

Sample identification	Gender	Age in months	Diagnosis	Assemblage	HAZ	WAZ	WHZ
188	M	42.6	Negative				
189	M	24.6	Negative		-0.66	-0.17	0.56
190	M	42.0	*Giardia lamblia*	B	-1.19	0.09	1.04
191	M	34.7	*Giardia lamblia*	B	-2.53	-1.15	0.44
192	F	25.4	Negative		-0.20	-1.11	-1.20
193	M	45.2	Negative		-1.38	-0.43	0.50
195	F	33.9	Negative		-2.54	-0.22	1.75
196	F	21.7	Negative		No	-0.94	-0.18
198	F	27.9	*Giardia lamblia*	A/E	-1.44	-0.61	0.46
199	M	29.2	Negative		-0.38	-0.41	-0.20
200	M	35.2	*Giardia lamblia*	B	0.64	1.02	0.87
201	F	34.5	*Giardia lamblia*	B			
202	M	37.7	Negative		-0.54	-0.45	-0.28
203	F	33.3	*Giardia lamblia*	B			
204	F	27.9	*Giardia lamblia*	B	-0.90	-0.14	0.62
206	F	37.5	*Entamoeba coli*		-1.22	-0.72	0.00
207	M	32.2	Negative		-0.95	-0.68	-0.18
208	F	52.8	Negative		-0.78	1.30	2.33
209	F	43.0	Negative		-0.46	0.05	0.52
211	M	20.6	*Giardia lamblia*<br>*Endolimax nana*	A	No	1.02	1.04
212	M	38.9	*Giardia lamblia*	A			
213	F	33.9	Negative		-1.59	-0.37	0.82
214	F	21.7	Negative		2.48	0.97	-0.37

Hatched areas mean data not obtained. F: female; HAZ: height-for-age; M: male; No: value not obtained by the software; WAZ: weight-for-age; WHZ: weight-for-height.
